# High Power Cathodes from Poly(2,2,6,6-Tetramethyl-1-Piperidinyloxy Methacrylate)/Li(Ni_x_Mn_y_Co_z_)O_2_ Hybrid Composites

**DOI:** 10.3390/polym13060986

**Published:** 2021-03-23

**Authors:** Guillaume Dolphijn, Fernand Gauthy, Alexandru Vlad, Jean-François Gohy

**Affiliations:** 1 Institute of Condensed Matter and Nanosciences (IMCN), Université Catholique de Louvain, Place L. Pasteur 1, B-1348 Louvain-la-Neuve, Belgium; guillaume.dolphijn@solvay.com (G.D.); alexandru.vlad@uclouvain.be (A.V.); 2 Solvay S.A., R&I Solid State Battery Applicability (SSBA) Laboratory, Rue de Ransbeek 310, 1000 Brussels, Belgium; fernand.gauthy@solvay.com

**Keywords:** redox polymers, NMC, hybrid materials, Li-ion batteries, high power, pseudo-capacitors

## Abstract

Lithium-ion batteries are today among the most efficient devices for electrochemical energy storage. However, an improvement of their performance is required to address the challenges of modern grid management, portable technology, and electric mobility. One of the most important limitations to solve is the slow kinetics of redox reactions associated to inorganic cathodic materials, directly impacting on the charging time and the power characteristics of the cells. In sharp contrast, redox polymers such as poly(2,2,6,6-tetramethyl-1-piperidinyloxy methacrylate) (PTMA) exhibit fast redox reaction kinetics and pseudocapacitors characteristics. In this contribution, we have hybridized high energy Li(Ni_x_Mn_y_Co_z_)O_2_ mixed oxides (NMC) with PTMA. In this hybrid cathode configuration, the higher voltage NMC (ca. 3.7 V vs. Li/Li^+^) is able to transfer its energy to the lower voltage PTMA (3.6 V vs. Li/Li^+^) improving the discharge power performances and allowing high power cathodes to be obtained. However, the NMC-PTMA hybrid cathodes show an important capacity fading. Our investigations indicate the presence of an interface degradation reaction between NMC and PTMA transforming NMC into an electrochemically dead material. Moreover, the aqueous process used here to prepare the cathode is also shown to enable the degradation of NMC. Indeed, once NMC is immersed in water, alkaline surface species dissolve, increasing the pH of the slurry, and corroding the aluminum current collector. Additionally, the NMC surface is altered due to delithiation which enables the interface degradation reaction to take place. This reaction by surface passivation of NMC particles did not succeed in preventing the interfacial degradation. Degradation was, however, notably decreased when Li(Ni_0.8_Mn_0.1_Co_0.1_)O_2_ NMC was used and even further when alumina-coated Li(Ni_0.8_Mn_0.1_Co_0.1_)O_2_ NMC was considered. For the latter at a 20C discharge rate, the hybrids presented higher power performances compared to the single constituents, clearly emphasizing the benefits of the hybrid cathode concept.

## 1. Introduction

Batteries and supercapacitors both rely on electrochemical processes that determine the power and energy performance metrics. Whereas Li-ion batteries (LIBs) rely on bulk reaction properties, supercapacitors, also known as electrical double layer capacitors (EDLCs), involve surface charge storage. Although significant progress has been made to provide higher power density batteries and higher capacity density supercapacitors, research is also focusing on electrode materials that combine the best of both worlds. Recognizing that certain electrode materials can display EDLC-like power performances while featuring redox processes that bring their capacity closer to traditional LIB materials, a new classification was proposed for them as pseudocapacitive materials [1]. The combination of a (pseudo)capacitive component and a faradaic component has been explored but the electrochemical response of such hybrid systems appears to be the sum of each separate component [2]. The main drawback of such a hybrid approach is thus the absence of synergy between the active materials. At low current density, the LIB material is the main contributor to the stored energy whereas the EDLC component can be seen as dead weight lowering the energy density. At high current density, on the other hand, the response is dominated by the EDLC component while the LIB material presents a poor response. To solve these issues, a hybrid electrode with synergetic effect of both components is required. A common strategy involving different redox components with a synergetic effect can be found in the redox mediator also known as the redox shuttle effect. In such a system, small organic molecules capable of rapid charge transfer are dissolved in the electrolyte or grafted onto the electrode active material. Their main objective is to protect the active material from overcharge. Typically, under a high current intensity, the redox mediator absorbs the current load, or is oxidized, and transfers this energy to the electrode active material [3,4]. However, the redox mediator presents a low contribution to the total stored capacity of the electrode.

**Figure 1:**
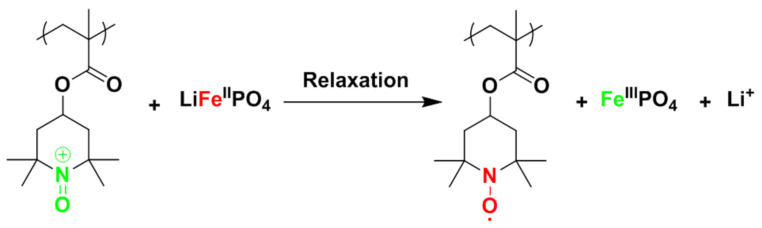
Redox process occurring in the cathode between the poly(2,2,6,6-tetramethyl-1-piperidinyloxy methacrylate) (PTMA) and the LiFePO_4_ (LFP) species. Green represents the oxidized species (charged form) and red represents the reduced species (discharged form).

At the crossroad between LIB and EDLC materials, a family of redox materials able to store charges by faradaic reaction exists but with a very fast charge transfer response. The poly(2,2,6,6-tetramethyl-1-piperidinyloxy methacrylate) (PTMA) redox polymer presents such characteristics and in addition, an excellent capacity retention for extended cycling [5]. Nevertheless, there is still a gap between nitroxide-based polymer electrodes and LIB materials regarding the specific energy density. A straightforward approach to reach higher capacity for such pseudocapacitive materials would be to combine PTMA with different high energy storage materials within a single device. Making synergistic use of pseudocapacitive and battery components, we previously showed that enhanced battery-capacitor hybrids offering more than the sum of their components can be realized by careful choice of the supercapacitive and battery materials [6]. This idea is exemplified by the choice of LiFePO_4_ (LFP) and PTMA as battery and pseudocapacitive materials, respectively. The key design principle of this concept is the rapid electrochemical response in combination with the higher working potential of PTMA. This allows, at equilibrium, the preferential charging of LFP at the expense of PTMA. Nonetheless, PTMA is the fastest discharge component. Using an appropriate pulse charge sequence, the rapid response of PTMA ensures the fast charge. During the subsequent relaxation, PTMA charges the LFP component, as shown in Figure 1. Thus, in the electrode, PTMA plays the role of power buffer and LFP plays the role of energy tank.

Overall, this hybrid system was evidenced to provide high energy and power capacity, over-polarization protection, and fast and stable recharge over more than 1500 cycles [6]. Applying the same rationale, we next designed a system based on the coupling of PTMA with a higher voltage material, namely LiMn_2_O_4_ [7]. The aim here was to obtain hybrid electrodes with enhanced power delivery characteristics, as oxidized PTMA^+^ will be both the favored and the best rate capable redox species—hence acting as a power buffer. Indeed, we have demonstrated the intra-electrode energy transfer from LiMn_2_O_4_ towards PTMA. As a result, enhanced power delivery as well as longer cycle life were obtained for the hybrid cathode. 

The implementation of the hybrid concept for specific application dealing with electric vehicles requires studying the impact of hybridizing PTMA with the dominant cathode materials in this specific market, i.e., nickel-rich layered oxides Li(Ni_x_Mn_y_Co_z_)O_2_ (NMCxyz with x + y + z = 1). In this contribution, hybrid NMC-PTMA cathodes were thus fabricated and studied.

## 2. Materials and Methods

### 2.1. Materials

PTMA was synthesized by free radical melt polymerization and blended with 15 wt% of C65 (Timcall) as a conductive agent to lead to PTMA/C composite by upscaling a previously reported procedure [5]. 2,2,6,6-tetramethylpiperidin-4-yl-methacrylate (TMPM, TCI), C65 (Timcall), hydrogen peroxide 35% (Sigma Aldrich), sodium tungstate dihydrate (Sigma Aldrich), and ethylenediaminetetraacetic acid disodium salt (EDTA, Sigma Aldrich), were used as received. Azobisisobutyronitrile (AIBN, Sigma Aldrich) was purified via recrystallization in methanol before use. Ethylene glycol dimethacrylate (EGDME, Across Chemicals) was purified by column chromatography using basic alumina before use. For the poly(TMPM)/C (PTMPM/C) 100 g scale synthesis used here, 85 g TMPM, 15 g C65, 0.575 g AIBN, and 1.6 mL EGDME were homogeneously mixed and further heated at 80 °C for 12 h. For the oxidation of PTMPM/C into PTMA/C, 20 g of PTMPM/C were introduced in a flask with 400 mL of methanol, 4.64 g of Na_2_WO_4_·2H_2_O, 3 g of EDTA, and 50 mL of H_2_O_2_ 35%. The resulting mixture was heated up to 60 °C for 40 h. The obtained PTMA/C composite powder was filtered off the solution and washed with 400 mL of methanol and 2 times with 600 mL of water. Finally, the recovered powder was dried and ground for 1 h in a ball miller to obtain the PTMA/C1 composite powder ready to be used as cathode material. NMC523 (MTI xlt), NMC811 (Forge Nano), NMC811Al_2_O_3_ (Forge Nano), *N*-methyl pyrrolidone (NMP, Acros), poly(vinylene difluoride) (PVDF5130, Solvay), carboxymethyl cellulose (CMC, MTI xlt), and styrene-butadiene rubber latex (SBR, MTI xlt) were used as received and stored in an Ar-filled glovebox. One mole of lithium hexafluorophosphate in ethylene carbonate–dimethyl carbonate 1/1 vol% (Solvionic) was used as electrolyte.

### 2.2. Hybrid NMC–PTMA Cathode Preparation

A typical example of an aqueous-processed hybrid cathode preparation is given here. A combination of 840 mg of PTMA/C, 200 mg of C65, 6 g CMC 1.5 wt% aqueous solution, and 120 mg of SBR latex are added in a 30 mL vial and mixed with a magnetic stirrer (800 rpm) with glass beads for 2 h. Then, 840 mg of NMC is added and mixed for 10 min. The resulting slurry is deposited onto a 20 µm etched aluminum foil (JCC Co. Ltd., Seoul, South Korea) and cast using a doctor blade with a wet thickness of 150 µm (without Al foil). The electrode sheet is dried at 50 °C for 1 h at ambient atmosphere and further dried overnight at 70 °C under vacuum. The electrode is then calendered at 70 °C in an electric hot rolling press (MTI xlt, MSK-HRP-01) towards 50 µm (without Al foil).

Organic solvent-processed hybrid cathodes were typically obtained by mixing 15 wt% of PTMA/C, 70 wt% of NMC, 5% of PVDF5130, 5 wt% of C65, and 5 wt% of NMP. To obtain hybrid cathodes by the dry process, we mixed PTMA/C powder (40 wt%) with NMC (40 wt%) and conductive carbon (20 wt%) in a mortar and pressed the powder in order to obtain a pellet as an electrode.

### 2.3. Battery Testing

Circular cathodes of 15 mm in diameter were punched using a heavy-duty disk cutter (MTI xlt), vacuum-dried for 12 h at 70 °C, and transferred into an Ar-filled glovebox for coin cell assembly (CR2025). Lithium disks of 16 mm cut from lithium ribbons (Sigma Aldrich, 0.75 mm thick) were used as counter and reference electrodes. Glass microfiber filter disks (Whatman, GF/D, 16 mm diameter) were used as separators. For each cell, 130 µL of electrolyte. Coin cells were pressed at 1000 kg cm^−2^ using a hydraulic crimping machine (MTI xlt) and aged for 24 h before testing. Electrochemical measurements were performed on a Biologic VMP300. 

## 3. Results

**Figure 2:**
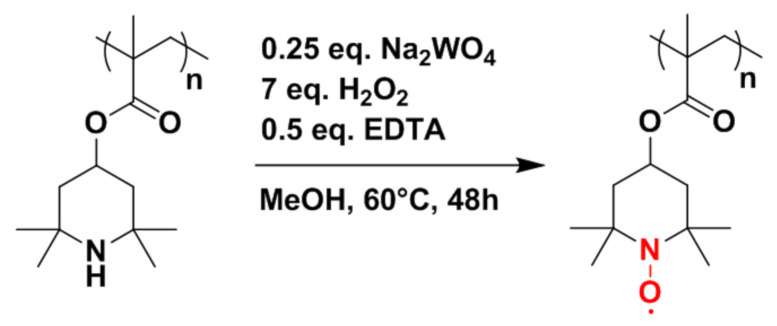
Oxidation reaction of PTMPM into PTMA.

Before considering hybrid electrodes composed of PTMA/C and NMC, the synthesis of PTMA/C composites should be briefly discussed. The synthetic pathway toward the production of the PTMA/C composite requires three steps: (i) the polymerization of the polymer hosting the carbon filler, (ii) the grinding step to obtain a thin powder, and (iii) the oxidation of the composite. In a first step, we focused our efforts on the polymerization step with the objective of evaluating a process for the production of the amine precursor composite material (PTMPM/C) at a 100 g scale (see chemical structure of PTMA and of its amine precursor PTMPM in Figure 2). Practically, the reagents were introduced in a container with glass beads and mixed for 4 h. After the mixing step, the beads were removed by sieving and the container containing the mixed reagents was heated to 80 °C (thus above the melting temperature of the TMPM monomer located at 60 °C) in a vacuum oven overnight to obtain the composite material with 98% yield. In a second step, 20 g of PTMPM/C was ground in a ball miller and the powder was oxidized in a third step using the conditions summarized in Figure 2. A small amount of crosslinker (EGDME) was added during the synthesis in order to obtain a polymer network. The addition of this crosslinker is mandatory since PTMA is known to exhibit some solubility in the battery electrolyte [5]. Therefore, capacity fading upon cycling would be observed if PTMA chains are diffusing in the electrolytes. One efficient way to avoid this drawback is to consider a PTMA network instead of linear PTMA chains. When immersed in the electrolyte, the PTMA network swelled but did not migrate out of the cathode, and further allowed a good ionic diffusion inside the cathode materials. In this respect, the counter anions (i.e., PF_6_^−^) from the electrolyte were able to diffuse through the particle in order to balance the positive charges formed during the oxidation of PTMA (charge process of the redox polymer leading to oxoammonium cations). The combination of the PTMA crosslinked network with a percolated structure of conducting carbon species in the PTMA/C composite is indeed a prerequisite to obtain high power electrochemical performances from our materials [5,6]. To illustrate this point, we synthesized the PTMA/C composite via a suspension polymerization approach (see the description and discussion of those results in the Supplementary Information section, Figures S1 and S2). The PTMA/C composite prepared by suspension polymerization showed much weaker electrochemical performances than the one prepared by bulk polymerization (Figure S3) although both samples displayed the same chemical composition (Figure S4). The poor electrochemical performances of the sample prepared by suspension polymerization are explained by the lack of percolated conducting carbon network in this sample (Figure S5). Adding a surfactant to improve the dispersion of conducting carbon inside the PTMA matrix was not successful (Figures S6 and S7). Therefore, the suspension polymerization approach was abandoned.

**Figure 3:**
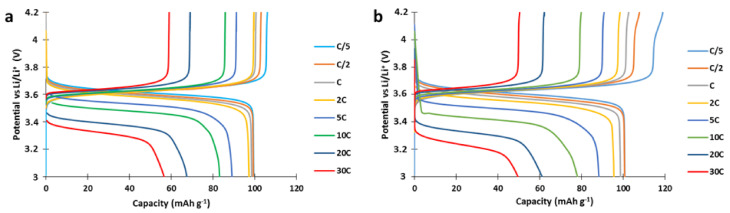
Voltage curves of the PTMA/C composite material cathode. (**a**) bulk synthesis as in ref. [5], (**b**) 100 g bulk synthesis.

To study the power performances, cathodes were produced and cycled with a C/5 charge current and variable discharge current (from C/5 to 30C) in a half-cell setup (Figure 3). The capacity delivered by the 100 g scale composite material synthesized here at low discharge rates was 8% lower than our previous results at small scale (92 here vs. 100 mAh g^−1^ in [5]). However, the power performances of the 100 g scale composite material at high C rate (e.g., 49 mAh g^−1^ at 30C, Figure 3) were quite good, indicating an efficient percolation of the carbon network in the composite particle and validating the utilization of the PTMA/C composite in hybrids with NMC. The discrepancy between the 100 g scale materials described here and the previously investigated small scale materials could originate from an incomplete oxidation of the PTMPM precursor.

**Figure 4:**
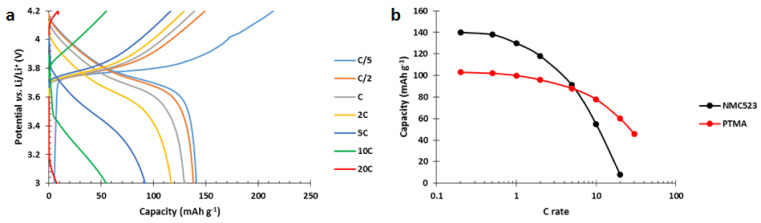
(**a**) Single constituent NMC523 cathode voltage profile at C/5 charge rate and variable discharge rates. (**b**) Capacity vs. C rate of NMC523 and PTMA/C cathode. The NMC523 cathode presents an area capacity of 0.6 mAh cm^−2^ and the PTMA/C an area capacity of 0.4 mAh cm^−2^.

To ensure that hybridizing NMC with PTMA/C composite in a cathode will increase the power performances, we must first prove that the composite presents better power performances than NMC. In this regard, single constituent electrodes of PTMA/C and Li(Ni_0.5_Mn_0.2_Co_0.3_)O_2_ (NMC523) were produced by aqueous process. They were cycled between 3 and 4.2 V vs. Li/Li^+^ at a constant charge rate (C/5) and variable discharge rate (from C/5 to 30C, Figure 4).

The NMC523 presents an initial capacity of 140 mAh g^−1^ (theoretical capacity ~155 mAh g^−1^) at C/5 discharge rate. As the discharge rate increases, the discharged capacity decreases to 8 mAh g^−1^ at 20C (Figure 4a). A PTMA-based cathode, in comparison, presents better power performances and outperforms NMC523 above 5C discharge rate (Figure 4b). This comparison illustrates the best power performances of PTMA/C compared to NMC, and confirms the interest of producing hybrid NMC-PTMA electrodes.

**Figure 5:**
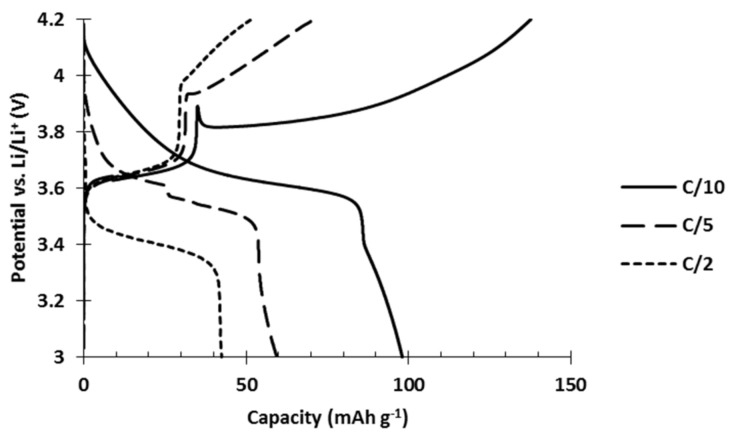
Voltage profile of a hybrid cathode made of NMC523 (42 wt%) and PTMA/C (42 wt%) made by aqueous process at different discharge rates.

Hybrid electrodes composed of PTMA/C (42 wt%) and NMC523 (42 wt%) were produced by an aqueous process. They were tested in order to evaluate their power performances compared to single constituent references. The results show neat inferior performances of the hybrid electrode (Figure 5). At low C rate, the initial capacity only reached 99 mAh g^−1^ while the addition of the respective capacities would lead to 120 mAh g^−1^. Furthermore, as the discharge rate slightly increased to C/5 and C/2, which are still low C rates in comparison to the 20C tested for the single constituent electrodes, a dramatic decrease of the capacity appeared. At C/2, only 42 mAh g^−1^ was recovered from the hybrid electrode while PTMA/C cathodes delivered 100 mAh g^−1^ and NMC523 cathodes delivered 136 mAh g^−1^.

**Figure 6:**
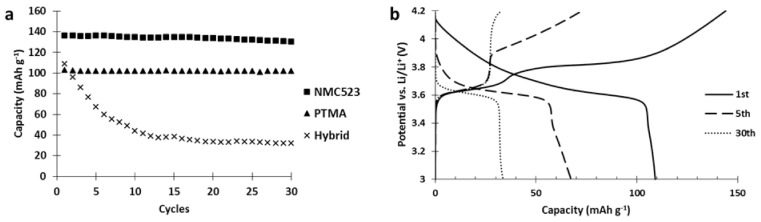
(**a**) Cycle life comparison between hybrid and single constituent electrode (charge and discharge at C/5). All electrodes have been made by aqueous processing. (**b**) Voltage profile of the 1st, 10th and 30th cycle of the hybrid NMC523-PTMA electrode.

Since the hybrid electrode was not reaching the performances of both the single constituent materials, a deterioration occurring between the materials was suspected. To investigate this issue, the cycle life of our hybrid electrode was tested and compared to the single constituent electrodes (Figure 6).

Results of the cycling tests (Figure 6) clearly show a strong capacity fading occurred in the hybrid electrode while it was absent in both single constituent electrodes. Moreover, analysis of the hybrid voltage curve (Figure 6) shows that upon cycling, the capacity stored by NMC523 gradually decreased until it vanished (charge curve, capacity between 3.7 and 4.2 V vs. Li/Li^+^) while the capacity stored by PTMA decreased by 10 mAh g^−1^ after 5 cycles and remained stable (capacity below 3.7 V vs. Li/Li^+^). This behavior suggests that PTMA acts on the NMC523 and transforms it into an electrochemically dead material. Figure 5 also shows that the NMC523 component was seriously affected while the PTMA kept its initial capacity, and was even more pronounced when the amount of PTMA in the hybrid cathode was increased (Figure S8). This suggests that PTMA speeds up the deterioration of the NMC. Interestingly enough, this degradation is specific to the NMC-PTMA combination in the hybrid cathode since neither LiCoO_2_-PTMA (Figure S9) nor LiNi_0.5_Mn_1.5_O_4_-PTMA (Figure S10) hybrid cathodes showed the important capacity fading observed for NMC-PTMA cathodes.

**Figure 7:**
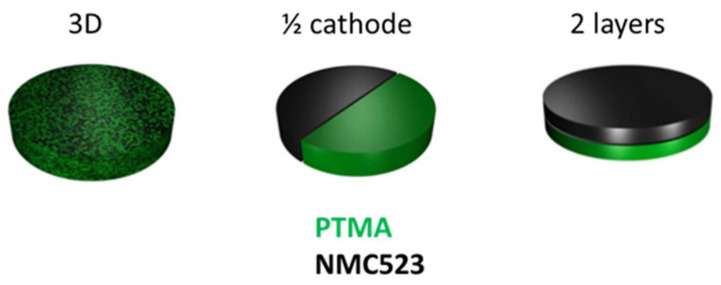
Hybrid NMC-PTMA electrode design.

Since the PTMA and NMC were homogeneously mixed in our hybrid electrode, it was interesting to investigate if this deterioration occurred due to contact between the materials or by secondary reactions occurring via the electrolyte. To investigate this question, three hybrid electrode designs were studied (Figure 7). The 3D design was the common hybrid electrode with the two components homogeneously mixed. The ½ cathode design was composed of two half cathodes made of one component so that the active materials were not in contact. The 2 layers design was an intermediate between the two previous designs where two layers of single constituent superpose themselves. In this design, the active materials were in contact but the surface of contact was much lower than in the 3D design.

**Figure 8:**
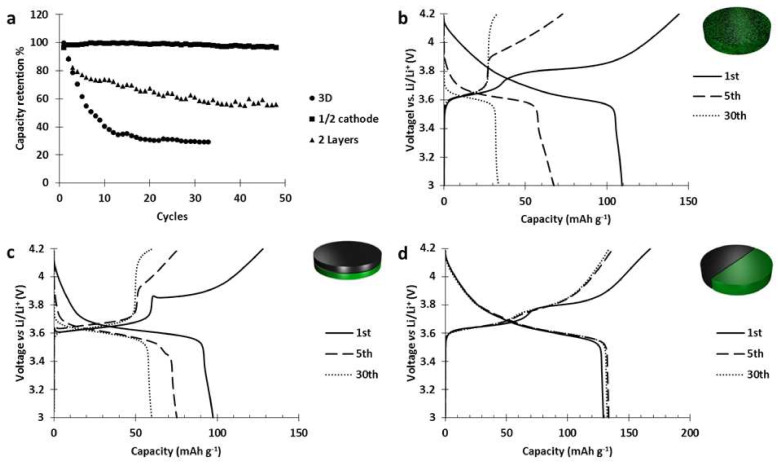
(**a**) Capacity retention of the hybrid electrode designs. Voltage curves of the 1st, 5th, and 30th cycle of the (**b**) 3D design, (**c**) two layers design, (**d**) half electrode design.

Those electrodes were cycled at C/5 and the results (Figure 8) showed that when there is no contact between the materials (1/2 cathode design), we do not observe any degradation. Moreover, when the materials are in contact (3D and 2 layers designs), the decrease of the contact surfaces slowed down the deterioration but could not prevent it.

Thus, in the 2 layers design (Figure 8c) after 30 cycles, the capacity stored by the NMC layer almost disappeared while the activity of PTMA, after decreasing of 10 mAh g^−1^, remained intact. Those results prove that the deterioration of the NMC523 was catalyzed by the PTMA component and occurred at the interface between PTMA and NMC.

**Figure 9:**
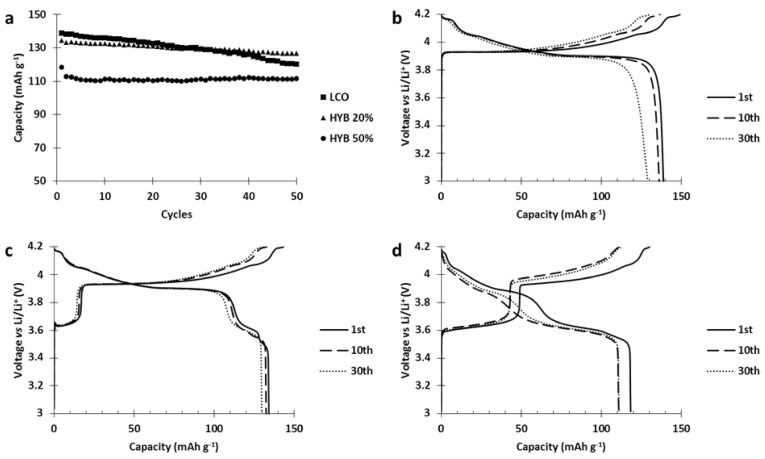
(**a**) Capacity retention through cycles of hybrid cathodes made by aqueous, *N*-methyl pyrrolidone (NMP)-based, and dry processes. (**b**) Voltage curves of the hybrid cathode made by (**b**) NMP-based process, (**c**) dry process, and (**d**) aqueous process.

Since water can deteriorate the inorganic component of a cathode, we studied its impact on the deterioration reaction between PTMA and NMC523. To this end, hybrid cathodes were produced by an NMP-based process and by a dry process and the capacity retention was compared with an aqueous-based process. Those two cathodes were cycled at C/5 between 3 and 4.2 V vs. Li/Li^+^ and their capacity retentions were compared with a hybrid cathode (15 wt% of PTMA/C, 69 wt% NMC523) produced by an aqueous process (Figure 9). The comparison of the capacity retention for the three cathodes (Figure 9a) clearly highlights that a huge capacity drop only occurred in the hybrid cathode made by aqueous process while the two other hybrid cathodes presented a relatively stable capacity retention through cycles. This demonstrates that water in the manufacturing process was at the origin of the deterioration between PTMA and NMC523. Moreover, since the interfacial contact between PTMA and NMC was present in all three cathodes, it implies that the surface of NMC was, somehow, activated by water. Afterwards, this activated surface reacted with PTMA through cycling and made the NMC particles electrochemically inactive.

The NMC523 slurry presents a highly alkaline pH of 11.3 (Figure S11). To reach such a high pH, alkaline species must have dissolved into water from the surface of the inorganic particle. It is well-known that LiOH is formed at the surface of NMC523 particles in contact with water [8,9] and corrodes the aluminum current collector onto which the aqueous cathode slurry is deposited [9]. A part of the lithium contained at the NMC surface was consumed by this reaction induced by contact with water. This lithium leaching implies a charge compensation for the loss of Li^+^ in the near surface region. One possibility is the electrochemical delithiation analogous to the charging process, where nickel ions are oxidized [10,11], but since the oxidation of nickel is not thermodynamically favored [12], other authors consider that Ni^3+^ is spontaneously reduced into Ni^2+^ causing delithiation and loss of oxygen [10,13,14]. A last option would be a cation exchange where protons take the place of lithium in the layered oxide [10,15,16]. Finally, even though the mechanism is not perfectly understood, a clear correlation between the amount of nickel in the layered oxide and the amount of surface species (hydroxide and carbonates) was demonstrated [17]. This last correlation is useful to distinguish the electrochemical performances of NMC in water compared to NMP. As NMC is rich in nickel, its surface is more prone to delithiation in contact with water. As it becomes delithiated, the rhombohedral crystal structure is less stable and tends to transform into a more stable one (spinel or cubic phase). Moreover, when the particle undergoes a charging process, more lithium is extracted from the Li-deficient surface which favors even more the phase transition. In other words, after a charge, the surface of a NMC particle processed in an aqueous slurry is composed of less lithium than a NMC processed in NMP. In a sense, the surface of a NMC processed in water presents a state of charge higher than a NMC processed in NMP. In that respect, charging a NMC processed in water could be seen as charging a NMC at higher voltage (more lithium extraction) which is known to be detrimental for the cycling stability of NMC due to irreversible surface phase transition towards a cubic phase [18]. The formation of an ion blocking cubic phase on the surface of NMC inhibits the motion of Li^+^ ions and increases the charge transfer resistance [18]. Such NMC deterioration could be similar to what we observed when a hybrid NMC523-PTMA cathode processed in water is cycled. The polarization of the NMC component increased until the cut-off potential of 4.2 V vs. Li/Li^+^ was no longer sufficient to charge the particle. If this deterioration mechanism is the one occurring in our hybrid electrodes, it would mean that the PTMA-NMC523 interface increased the phase transition process. This hypothesis is meaningful to explain the huge capacity fading observed in the hybrid NMC523-PTMA cathode processed in water.

**Figure 10:**
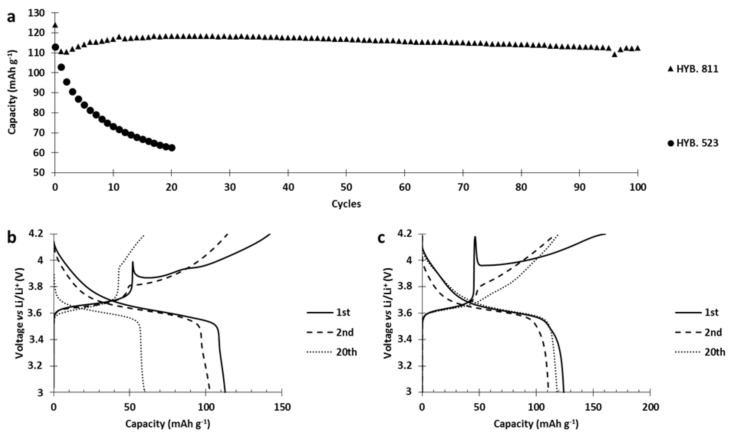
Hybrid NMC-PTMA cathodes cycled at C/5 with different NMC materials (NMC811, NMC53). (**a**) Discharged capacity through cycles. Voltage curves of the (**b**) HYB. 523, (**c**) HYB. 811.

In the next step, we replaced NMC523 with NMC811 in the PTMA/C–NMC hybrid cathodes. Figure 10a pinpoints the fact that the hybrid cathode based on NMC811 (referred to as HYB. 811) revealed a much better capacity retention than the hybrid based on NMC523 (referred to as HYB. 523). In order to explain this huge difference in capacity retention, we analyzed the capacity retention as well as the voltage curves (Figure 10). During the different charges of the HYB. 523 (Figure 10b), the capacity stored by PTMA (below 3.8 V vs. Li/Li^+^) decreased by 10 mAh g^−1^ between the 1st and 20th cycle, probably due to the loss of nitroxides that reacted with the NMC523 surface. The capacity stored by NMC523 (above 3.8 V vs. Li/Li^+^) decreased with cycles. Moreover, the 1st charge plateau of NMC523 started at 4 V and decreased sharply to 3.85 V vs. Li/Li^+^ (hereafter referred to as spike behavior) to finally follow the expected charge profile up to 4.2 V vs. Li/Li^+^. The second charge plateau did not present this spike and started at 3.81 V vs. Li/Li^+^. Through cycling, a gradual polarization of the charge of NMC523 took place due to reaction with PTMA, and finally the plateau started at 3.95 V vs. Li/Li^+^ for the 20th charge. For the HYB. 811 (Figure 10c), the capacity stored by PTMA increased by 10 mAh g^−1^ between the 1st and the 20th cycle (inflexion point at 3.7 V vs. Li/Li+) indicating no loss of electro-active nitroxide groups. The capacity stored by the inorganic component remained stable between the 2nd and 20th cycle. The first charge presents a strong spike behavior, at the beginning of the NMC811 plateau, from 4.17 V to 3.95 V vs. Li/Li^+^. As for NMC523, the spike was only present during the first charge. However, the following charge plateaus of the inorganic component gradually presented a lower polarization as it started at 3.75 V for the second charge and 3.71 V vs. Li/Li^+^ for the 20th charge. Since we do not observe an increase of polarization or a loss of capacity from both components in hybrid configuration, we can conclude that the surface of NMC811 does not react detrimentally with PTMA. The presence of the spike suggests that an ionically blocking layer was formed on the NMC particles during the cathode manufacturing. This layer impeded lithium motion and/or the electrical conduction so that the charge of NMC did not start below a certain voltage (4 V for HYB.523, 4.18 for the HYB.811). When this voltage was reached (the top of the spike), the particle started its oxidation process and lithium ions left the inorganic particle. After the spike, the potential decreased. The most logical explanation is an increase of ionic conduction through the layer (mechanical degradation or increase of its ionic conductivity) probably caused by the lithium ions coming from the crystal structure of the particle or the oxidation of the layer. During the second charge, the spike behavior is no longer present, indicating the inactivity or destruction of the initial ionic/electronic resistance layer. The spike behavior, discussed earlier, is not due to the presence of PTMA but to the contact between NMC with water during the slurry preparation.

**Figure 11:**
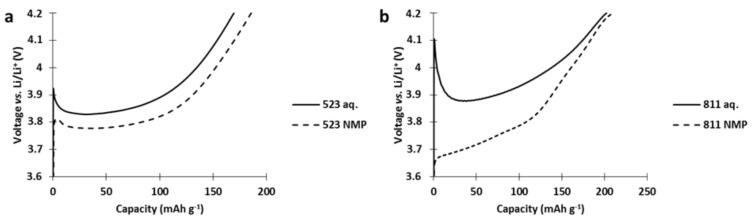
First charge profiles of (**a**) NMC523 and (**b**) NMC811 cathode produced from aqueous (aq.) and NMP-based slurry.

As presented in Figure 11, a spike appears directly at the beginning of the charge during the first charge of the single constituent NMC cathodes produced via aqueous process. This spike was not visible in the cathode fabricated from NMP slurries. In the literature, this spike behavior has already been observed for NMC523 immersed in water [8], or for Ni-rich cathodes stored for long periods in air [15,19,20], and is attributed to insulating species formed at the surface of the NMC particles upon aging.

**Figure 12:**
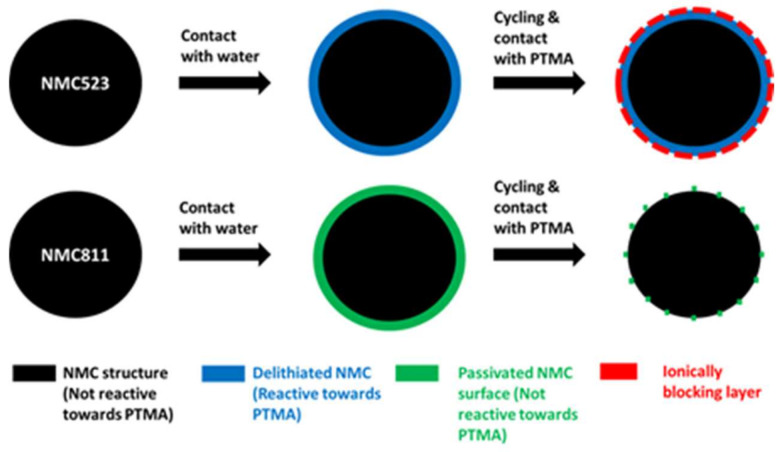
Schematic view illustrating the differences on the layer nature formed onto NMC523 and NMC811 during the aqueous processing and the evolution of this layer through cycling in contact with PTMA.

However, the spike behavior of NMC811 is more pronounced than for NMC523. We attribute this difference to the higher resistance of the layer formed onto the NMC811 particles. The nature of this layer, formed onto the particle during the slurry preparation, and its evolution through cycles could explain the unexpected stability of NMC811 in the hybrid electrode. In the scheme below (Figure 12), a model is presented which could explain the different behaviors. During the hybrid cathode fabrication, the surface of NMC523 was delithiated (in blue) and became reactive towards PTMA. During cycling, the interfacial reaction produced an additional resistance layer (in red). In the case of NMC811, the reaction with water was more important as illustrated by a higher rise of pH (pH of slurry containing NMC523 = 11.3, NMC811 = 12.1) and a stronger spike behavior. The surface reactivity produced a thin passivation layer (in green) onto NMC811. The formation of this passivation layer was evidenced by X-ray photoelectron spectroscopy (Figure S12) and was formed by Al-containing insoluble compounds resulting from the reaction of the Al current collector with alkaline species originating from the reaction of NMC811 with water. This passivation layer did not react with PTMA but was degraded through cycling (dotted green layer) and allowed transport of ions.

In this contribution, we previously attributed the increase of polarization and the capacity fading to the structural transformation of the surface of NMC523 from a spinel phase into an ionically blocking cubic phase. In our model, this transformation is illustrated by the blue layer (spinel) becoming a red layer (cubic). The nature of the passivation layer is not known. It could be a deposit of aluminate coming from the corrosion of the Al current collector due to the alkalinity of the slurry. Another possibility would be the high degree of surface delithiation due to the reaction with water transforming the NMC811 surface structure into a cubic phase that is not fatigue-resistant. Although we do not have X-ray diffraction results to support this model, this surface transformation and its magnitude likely have a big impact on the electrochemical properties of the NMC component in the hybrid cathode configuration. Moreover, our assumptions are strengthened by previous results reported in the literature in which the structural changes occurring at the surface of nickel-rich NMC were studied [21]. In the same direction, the production of a cubic phase (rock salt phase) on the subsurface of NMC exposed to moisture was previously reported [15]. Additionally, this reconstructed surface layer due to contact with air was seen to thicken if cycled [22].

**Figure 13:**
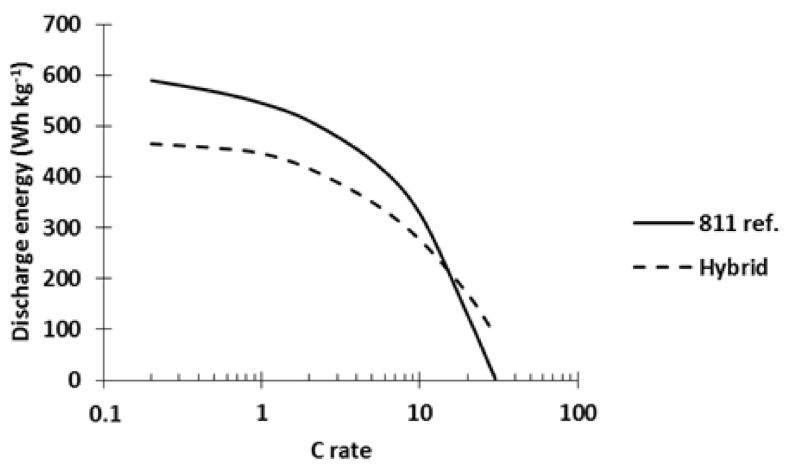
Discharge energy vs. discharge c rate of the NMC811_Al2O3_ reference cathode and the hybrid NMC811_Al2O3_–PTMA/C. Area capacity of both electrodes equal to 0.7 mAh cm^−2^. Charge rate equivalent to C/5. Both cathodes were made by aqueous process.

In order to prevent the reaction of water with the surface of NMC, we decided in a last step to study alumina-coated NMC [23,24]. We selected alumina-coated NMC811 (NMC811_Al2O3_) since NMC811 definely shows better performance than NMC523 (see above). The impact of blending NMC811_Al2O3_ cathode material with the PTMA/C composite on the power performances is depicted in Figure 13. A hybrid cathode made with 42 wt% of each active material and a reference NMC811_Al2O3_ cathode (811 ref.) were tested in coin cells, charged at C/5, and discharged at variable C rates. At low C rates, the 811 ref. cell delivered 27% more energy than the hybrid cathode (591 vs. 465 Wh kg^−1^, respectively). The energy delivered by the 811 ref. remained higher until 10C. At higher C rates, the hybrid cathode delivered more energy (at 20C: 47% more energy, see Figure 13). The results plotted in Figure 13 clearly demonstrate the better power performance of PTMA/C-NMC811_Al2O3_ hybrid cathodes compared to pure NMC811_Al2O3_ ones and further confirm the interest of the hybridization concept.

**Figure 14:**
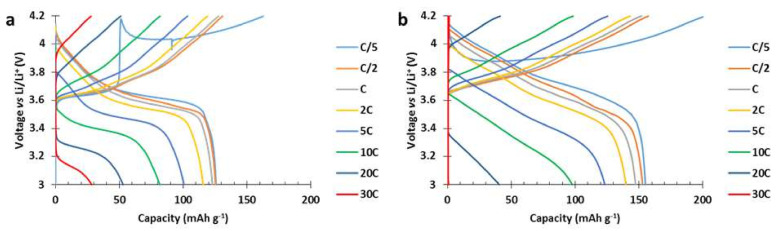
Voltage profiles of (**a**) the hybrid NMC811_Al2O3_-PTMA/C cathode and (**b**) the reference NMC811_Al2O3_ cathode. Areal capacity of both electrodes equal to 0.7 mAh cm^−2^. Charge rate equivalent to C/5. Both cathodes were made by the aqueous process.

The voltage profiles of each cathode are presented in Figure 14. At C/5 discharge rate, the 811 ref. delivered 155 mAh g^−1^ while the hybrid delivered 125 mAh g^−1^. At higher discharge rate, the redox process of the PTMA/C in the hybrid cathode enabled the potential of the cell to follow a flat plateau. This plateau was easily distinguishable from the discharge rates 2C until 30C (Figure 14a) while the 811 ref. (Figure 14b) presented a monotonic slope. Thanks to this plateau, the hybrid cathode delivered more capacity at 20C and 30C discharge rates compared to the 811 ref. The presence of this plateau also demonstrates the communication existing between the PTMA/C and NMC811_Al2O3_ components of the hybrid cathodes and further shows the improved power performance at high C rate thanks to the presence of PTMA/C.

**Figure 15:**
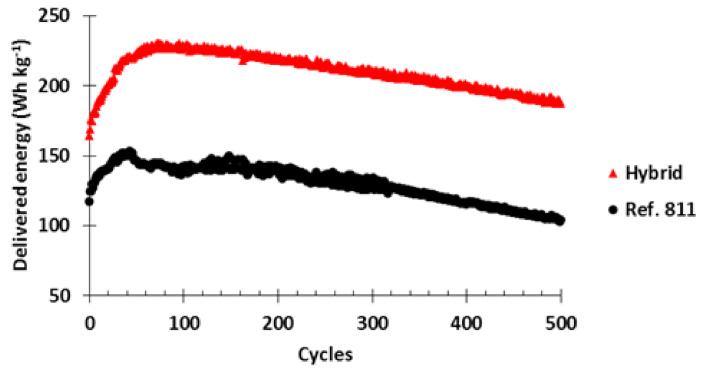
Delivered energy through cycles of half cells made with a hybrid NMC811_Al2O3_–PTMA/C cathode and the reference NMC811_Al2O3_ cathode. Charge current equivalent to C/5, discharge current equivalent to 20C.

Finally, the cycle life of both cathodes was monitored at C/5-20C charge-discharge rates and the results are presented in Figure 15. Initially, the hybrid cell delivered 40% more energy (164 Wh kg^−1^) than the 811 ref. (117 Wh kg^−1^) showing again the improved power performance of the hybrid cell. The delivered energy for both cells increased during the following cycles. After 37 cycles, the 811 ref. delivered 152 Wh kg^−1^ while the hybrid cell delivered after 71 cycles 230 Wh kg^−1^ (48% more than the maximum of 811 ref.). Afterwards, both cells presented an energy decay through cycles but the hybrid cell always showed a much higher energy than the pure NMC811_Al2O3_ one.

## 4. Conclusions

Hybrid cathodes were produced from aqueous slurries containing NMC and PTMA. The environmentally friendly aqueous processing of the cathodes was preferred compared to the organic solvent route because the latter generally results in cracked electrodes after the evaporation of the organic solvent and therefore poor reproducibility. However, a huge capacity fading of the aqueous based hybrid cathodes was observed. This degradation was attributed to a catalytic effect of the PTMA on the delithiated surface of NMC in the presence of water. It was shown that the capacity fading observed in the hybrid cathode is mainly correlated with the nature of the NMC used, being more pronounced for NMC523 than for NMC811. Those better performances were attributed to the most important reaction of NMC811 with water that passivated its surface and impeded the catalytic reaction with PTMA. Finally, alumina-coated NMC811_Al2O3_ cathodes were utilized for the fabrication of the hybrid NMC/PTMA cathodes in order to minimize degradation reactions. Compared to the single constituents, at a 20 °C discharge rate, the hybrids presented higher power performances as well as an extended cycle life clearly emphasizing the benefits of the hybrid cathode concept.

## Data Availability

The data presented in this study are available on request from the corresponding author.

## Supplementary Materials

The following are available online at https://www.mdpi.com/2073-4360/13/6/986/s1, Figure S1: Emulsion polymerization reactor where the synthesis of the PTMPM/C precursor is performed. Figure S2: (a) Composites granules collected after reaction without surfactants. (b) Optical microscopy picture of a granule. (c) Grinded granules showing a good homogeneity of the carbon in the polymer matrix. Figure S3: (a) Power performances of cathodes made with composite material PTMA/CSusp and PTMA/CBulk. (b) Voltage curves of PTMA/CSusp cathode. (c) and (d) Voltage curves of two different batches of PTMA/C bulk synthesized at small scale cathode (Bulk 1 and Bulk 2). Figure S4: TGA of the PTMA/CSusp and the PTMA/CBulk. Figure S5: Transmission electron microscopy pictures of a section of a PTMPM/C particle. (a) Low magnification picture with external part on the top right corner. (b) Magnification of the surface section with external part on the left. (c) Magnification of an inner zone between 18 and 27 µm from the surface. (d) Magnification of an inner zone above 30 µm from the surface. Figure S6: Extracted material from the suspension polymerization with Tween-20 as a surfactant. (a) Black granules of polymers and carbon. (b) Polymer without carbon. Figure S7: Extracted black composite granules and roughly grinded particles with a heterogeneous core-shell composition. Figure S8: (a) Comparison of the cycle life between a hybrid NMC-PTMA electrode made with 42 wt% and 15 wt% of PTMA/C. (b) Voltage profiles of a hybrid NMC-PTMA cathode cycled at C/5 (charge and discharge) with 15 wt% of PTMA/C and 69 wt% of NMC523 produced by aqueous process. Figure S9: (a) Capacity retention through cycles of LCO and hybrid LCO-PTMA cathodes at C/5 (charge and discharge). (b) Voltage curves of LCO. (c) Voltage curves of Hybrid 20%. (d) Voltage curves of Hybrid 50%. Figure S10: (a) Capacity retention through cycles of the LNMO reference and the hybrid cathodes cycled at C/5 (charge and discharge). (b) Voltage curves of the LNMO reference. (c) Voltage curves of the hybrid cathode. Figure S11: The pH of the different slurries monitored after 10 min of mixing. The slurries are made with 84 wt% of intercalation materials, 3 wt% SBR, 3 wt% CMC and 10 wt% C65. Figure S12: XPS spectra of Al_2p_ for (a) NMC523 and (b) NMC811. TEM images of (c) NMC523 and (d) NMC811.
