# Supplementary material for: High Power Cathodes from Poly(2,2,6,6-Tetramethyl-1-Piperidinyloxy Methacrylate)/Li(NixMnyCoz)O2 Hybrid Composites
Source: Polymers (Basel). 2021 Mar 23;13(6):986. doi: 10.3390/polym13060986 (PMC8004871; doi:10.3390/polym13060986)
Supplement: Supplementary file 1 [file polymers-13-00986-s001.pdf]

Supplementary information section for:

High power cathodes from poly(2,2,6,6-tetramethyl-1-piperidinyloxy methacrylate)/$Li(Ni_xMn_yCo_z)O_2$ hybrid composites

Guillaume Dolphijn [1,2], Fernand Gauthy [2], Alexandru Vlad [1] and Jean-François Gohy [1,*]

[1] Institute of Condensed Matter and Nanosciences (IMCN), Université catholique de Louvain, Place L. Pasteur 1, B-1348 Louvain-la-Neuve, Belgium; guillaume.dolphijn@solvay.com (G.D.); alexandru.vlad@uclouvain.be (A.V.)

[2] Solvay S.A., R&I Solid State Battery Applicability (SSBA) Laboratory, Rue de Ransbeek 310, 1000 Brussels, Belgium; fernand.gauthy@solvay.com

* Correspondence: jean-francois.gohy@uclouvain.be

1. Synthesis of PTMA/C composites by suspension polymerization

The objective of this section is to evaluate the possibility to obtain a thin powder (~20µm) of the precursor composite material (PTMPM/C), with an efficient conductive carbon network, ready to be oxidized. The following results present the behavior of the reaction without surfactants and, further, with surfactants. The suspension polymerizationwas first realized with a 40 g batch of composite material (15 wt% carbon) without any surfactant in a 1 l reactor (Figure S1).

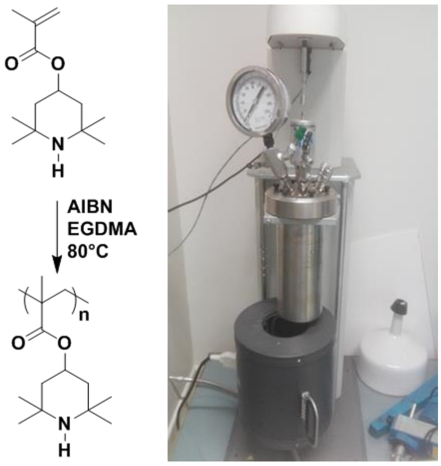

Figure S1. Emulsion polymerization reactor where the synthesis of the PTMPM/C precursor is performed.

The monomer, acetylene black (AB), ethylene glycol dimethacrylate (EGDMA) and AIBN were stirred in miliQ water at 300 rpm and, after the establishment of an inert atmosphere, the media was heated, up to 60°C in order to melt the monomer particles into droplets. After 30 minutes, the reactor was heated towards 80°C to start the polymerization. After 12h, the reaction was stopped and black composite granules (Figure S2) with a diameter between 2 and 5 mm were collected (yield = 93%) from a clear aqueous media. A rough milling shows that the entire particle is black (Figure S2c) which illustrates that the carbon material is well incorporated in the monomer droplets rather than being excluded to the interface with the water phase.

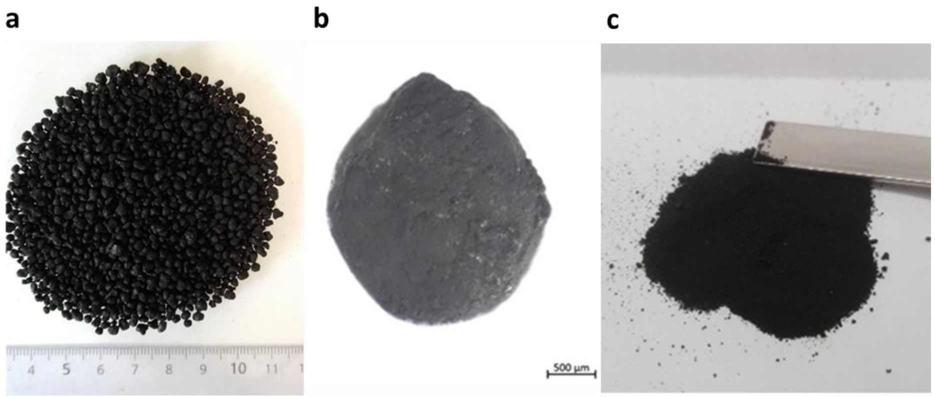

Figure S2. (**a**) Composites granules collected after reaction without surfactants. (**b**) Optical microscopy picture of a granule. (**c**) Grinded granules showing a good homogeneity of the carbon in the polymer matrix.

The granules were grinded in a ball miller to obtain a fine powder (~20µm) and 20 g of this powder were oxidized with the typical H_2O_2/Na_2WO_4 procedure to obtain the targeted composite material PTMA/C_{Susp}.

The electrochemical performances of the PTMA/C_{Susp} material were evaluated and compared with composite materials obtained by the bulk polymerization (PTMA/C_{Bulk}) at small scale described in our main manuscript. To perform this evaluation, cathodes made with 84 wt% active material, 3 wt% CMC, 3 wt% SBR and 10 wt% C65 were produced and assembled in a coin cell setup with a lithium disc as a counter electrode. The cells were charged at a C/5 current rate and discharged at variable rates (from C/5 to 30C) (Figure S3).

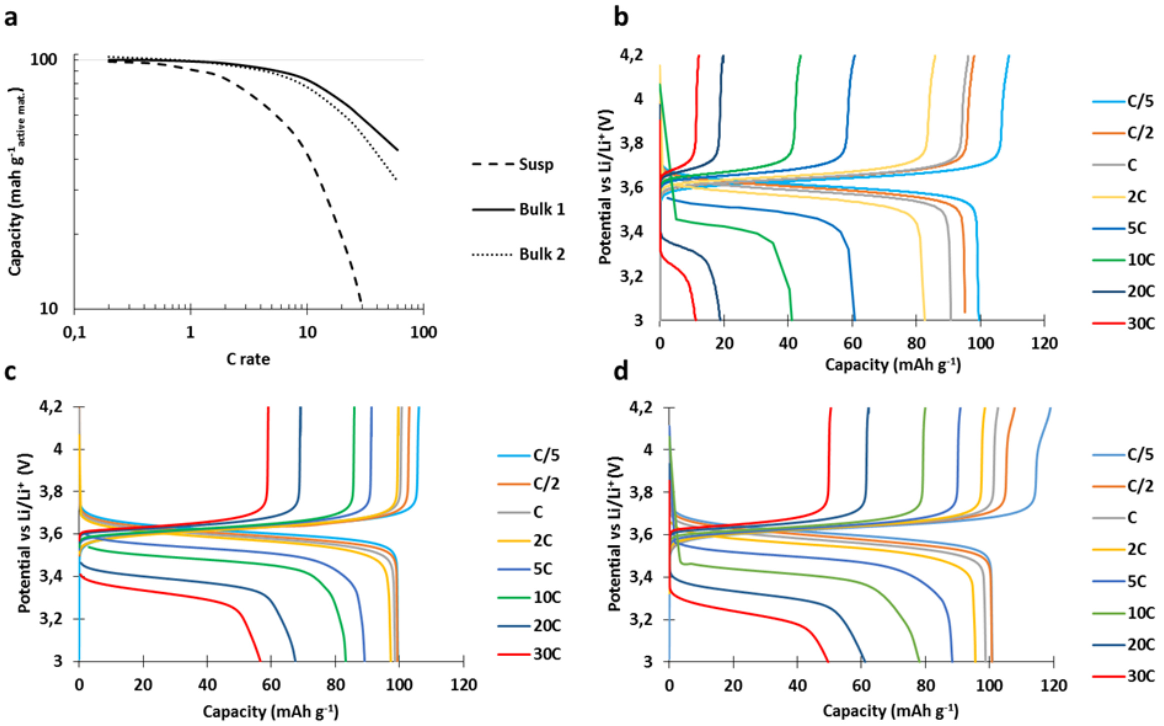

Figure S3. (**a**) Power performances of cathodes made with composite material PTMA/CSusp and PTMA/CBulk. (**b**) Voltage curves of PTMA/CSusp cathode. (**c**) and (**d**) Voltage curves of two different batches of PTMA/C bulk synthesized at small scale cathode (Bulk 1 and Bulk 2).

All cathodes release approximately the same capacity (~100 mAh g^{-1}) at low rates (C/5) meaning that there is the same amount of active material in the tested samples. On the other hand, the power performances of the PTMA/C_{Susp} material are significantly lower than the composite material obtained from a bulk polymerization (capacity at 30C: 11 for PTMA/C_{Susp}, 57 for PTMA/C_{Bulk1} and 50 for PTMA/C_{Bulk2}). Thermogravimetric analysis (Figure S4) confirms the presence of the same amount of carbon in each sample (15 wt% for the suspension composite vs. 16 wt% for the bulk composites). This suggests that the carbon percolation in the composite material made by suspension polymerization is less efficient.

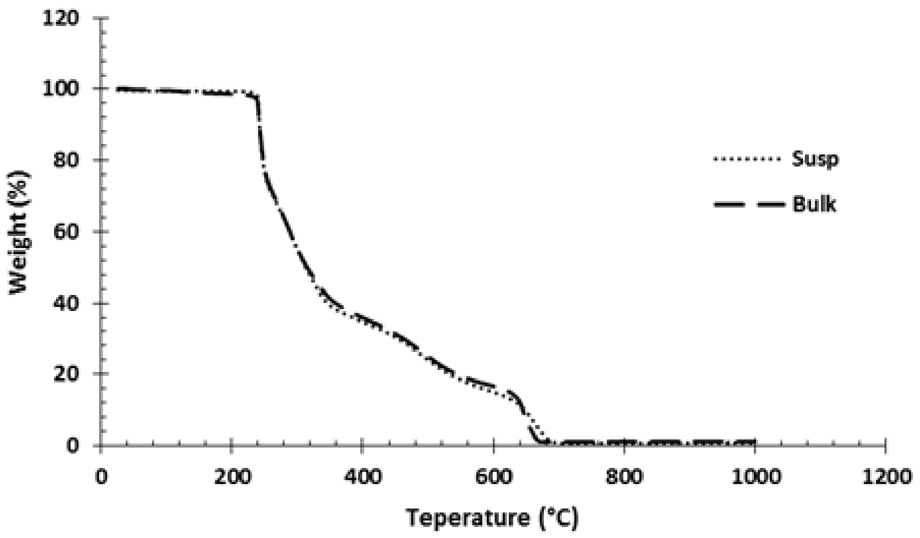

Figure S4. TGA of the PTMA/CSusp and the PTMA/CBulk.

The granules obtained from the suspension polymerization were analyzed by transmission electron microscopy (TEM) to observe the carbon percolation network (Figure S5). TEM images were obtained with a LEO 922 OMEGA energy filter transmission electron microscope. The low magnification picture of the cross section (Figure S5a) shows the presence of 3 regions: (i) a dark region from the surface towards ~20 µm deep; (ii) a region with holes leading to a white color between 20 µm and 25 µm deep and (iii) a light gray region above 30 µm deep. Each region was observed through high magnification. The near-surface region (Figure S5b) presents a high concentration of carbon homogeneously dispersed with a small amount of aggregates (black objects). Deeper, the region corresponding to the white layer presents a high number of holes. Their presence is attributed to aggregates that were removed during the sample preparation by ultramicrotomy. Deeper inside the particle (>30 µm deep, Figure S5d) a lower density of carbon particles is present leading to a light gray color.

Thus, even though the granules seem homogeneous at a macroscopic level, TEM measurement shows a heterogeneous dispersion of carbon particles inside. Moreover, the presence of carbon aggregates indicates that the shearing during the reaction is not sufficient to break them into small particles. As a result, grinding those granules leads to a heterogeneous powder composed of composite particles with a high or poor density of conductive carbon as the particle comes from the outer or the inner core of the granule, respectively. Since the particles with a poor carbon network cannot support high current loads during battery operation, the resulting power performances of the composite are lower than the one obtained from bulk polymerization where the homogenization of the different components is performed in a ball miller. Despite the lower power performances presented, the suspension polymerization without surfactants already gives composite granules in high yield validating the potential use of this technique to produce our

composite material. To optimize the process through the production of a fine powder, the introduction of a surfactant has been studied next.

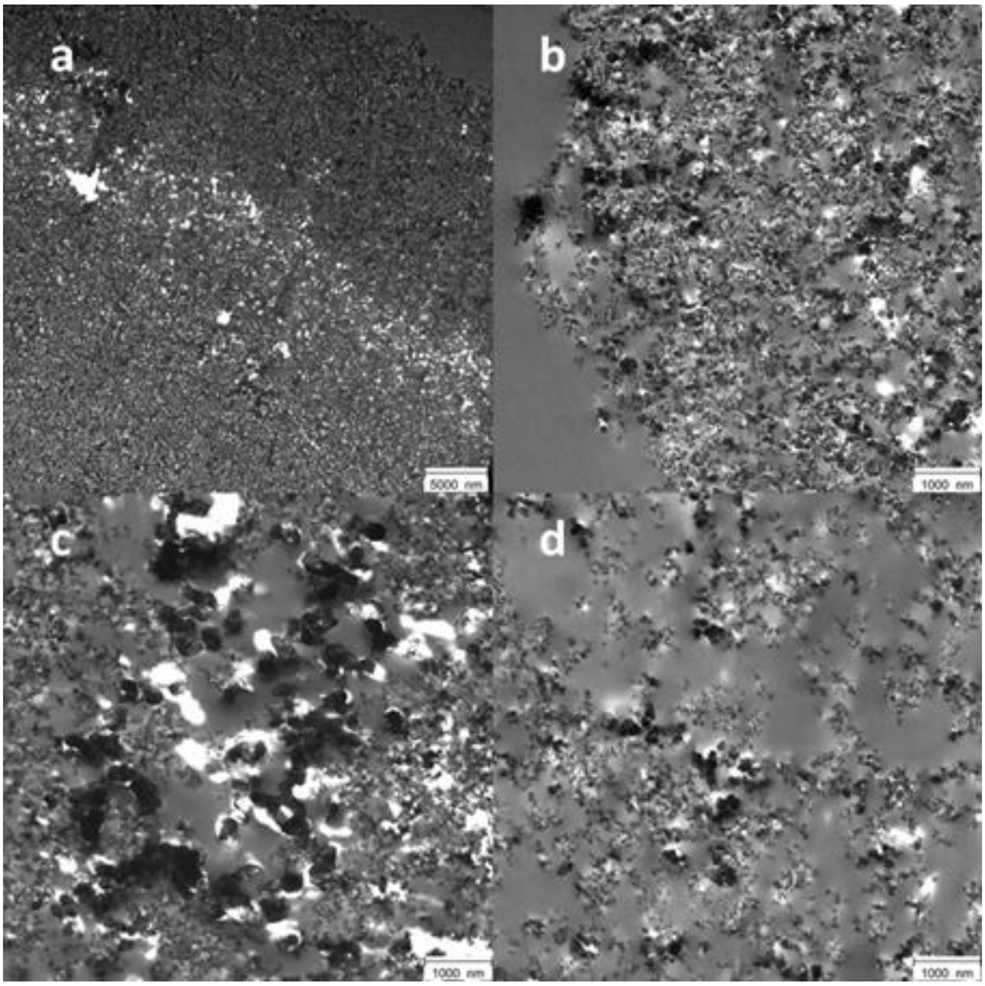

Figure S5. Transmission electron microscopy pictures of a section of a PTMPM/C particle. (**a**) Low magnification picture with external part on the top right corner. (**b**) Magnification of the surface section with external part on the left. (**c**) Magnification of an inner zone between 18 and 27 μm from the surface. (**d**) Magnification of an inner zone above 30 μm from the surface.

In a next step, we have studied the impact of the Polysorbate Tween-20 used as a surfactant for suspension polymerization. We conducted suspension polymerization attempts on 40 g batches, such as presented in the previous section, with surfactant concentrations varying between 10^{-4} and 10^{-2} mol l^{-1} but never obtained polymers. To study the impact of surfactants, we reduced the batches to 4 g and performed the reaction in the same condition in a round-bottom flask with a magnetic stirrer. The polymerization reaction succeeded and we extracted two distinct materials: black granules and a light gray solid (Figure S6).

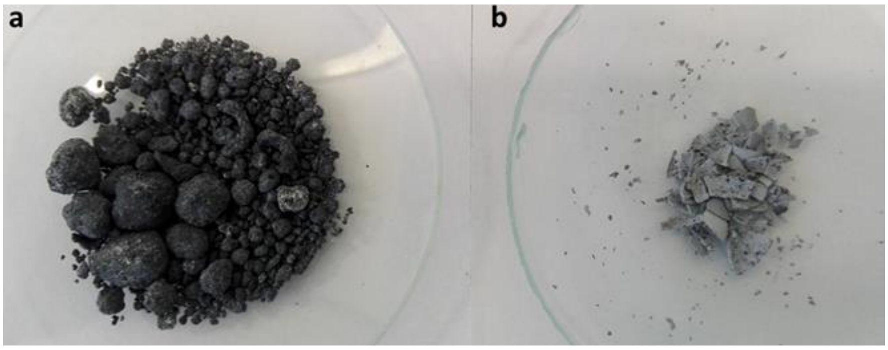

Figure S6. Extracted material from the suspension polymerization with Tween-20 as a surfactant. (**a**) Black granules of polymers and carbon. (**b**) Polymer without carbon.

The insoluble gray solid was attributed to cross-linked polymer with a small amount of carbon, and the black granules to the targeted composite material. The granules were roughly grinded to analyze the homogeneity of the whole particle and a core-shell structure was observed with a polymer core and carbon in the outer part (Figure S7).

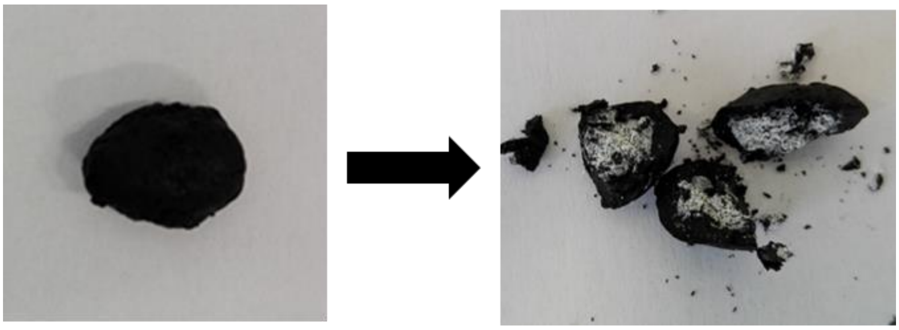

Figure S7. Extracted black composite granules and roughly grinded particles with a heterogeneous core-shell composition.

The use of Tween-20 during the suspension polymerization induces a phase separation between the monomer and the conductive carbon. Such phenomenon could result from the adsorption of Tween-20 at the surface of the carbon black. The carbon black is then covered by hydrophilic groups and has a tendency to phase separate from the monomer. As a result, the majority of the carbon is placed around the monomer droplets forming a core-shell particle after polymerization. Moreover, the smallest droplets polymerize without incorporation of carbon resulting in precipitation of pure polymer as a gray solid. The Tween-20 addition is detrimental for the homogeneity of the composite material and is, therefore, not an option to obtain thin composite particles.

2. Investigations on the origin of the capacity fading in PTMA/C-NMC hybrid cathodes

2.1. Effect of the amount of PTMA

A huge capacity fading was observed only on the hybrid electrode (Figure 5 in the main manuscript) while the single constituent electrodes revealed a classical cycling stability (Figure 4 in the main manuscript). These observations suggest a detrimental interaction between the PTMA and NMC523 active materials. It was also shown that the NMC523 component was seriously affected while the PTMA kept its initial capacity. This suggested that PTMA should speed up the deterioration of the NMC. To illustrate this fact, we have cycled a hybrid electrode with a lower amount of PTMA. In order to check a possible correlation between the amount of PTMA in the hybrid and the degradation level, two hybrids with different PTMA contents were tested (Figure S8). Both hybrids revealed a huge capacity drop upon cycling. **Error! Reference source not found.**a shows that even with 15 wt% of PTMA/C in the hybrid electrode, an important deterioration of

the NMC component occurs. After 50 cycles, only 15 mAh g^{-1} is stored by the NMC component during the charge while the capacity stored by PTMA remains stable (Figure S8b). Since more NMC is present in this hybrid electrode (69 wt% instead of 42 wt%), it takes more cycles to degrade the majority of the NMC and to stabilize the capacity delivered around 30 mAh g^{-1}. This indicates that the degradation is a continuous process occurring throughout the charge/discharge cycles and even a small amount of PTMA can deactivate a high amount of NMC.

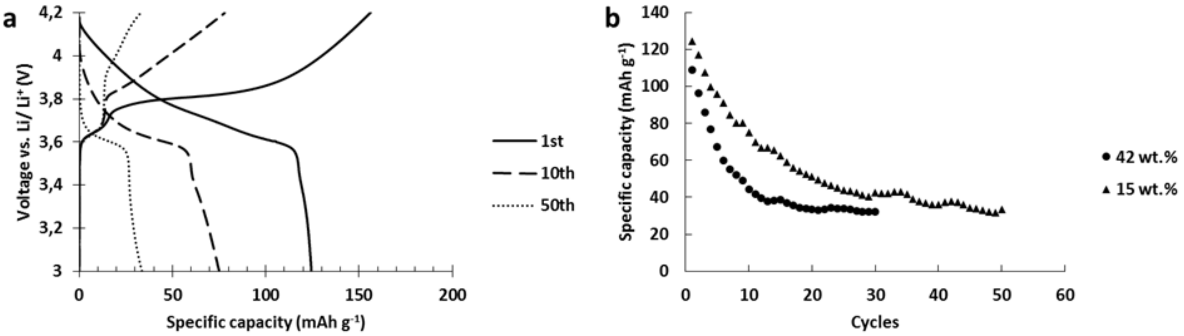

Figure S8. (**a**) Comparison of the cycle life between a hybrid NMC-PTMA electrode made with 42 wt% and 15 wt% of PTMA/C. (**b**) Voltage profiles of a hybrid NMC-PTMA cathode cycled at C/5 (charge and discharge) with 15 wt% of PTMA/C and 69 wt% of NMC523 produced by aqueous process.

2.2. Effect of the presence of nickel and cobalt in the intercalation material

In order to observe the correlation between the presence of cobalt in a layered oxide crystalline structure and the observation of huge capacity drop, $LiCoO_2$ (LCO), another layered oxide, was tested. Hybrid cathodes of PTMA/C and LCO were produced by aqueous processing with 50 wt% (HYB 50%) and 20 wt% (HYB 20%) of PTMA/C. As a reference, a single constituent cathode made of LCO was produced in the same way. They were cycled at C/5 in half cell set up in order to observe any degradation similar to the one observed with hybrid NMC cathode (Figure S9). Figure S9a shows that the capacity of the HYB 50% or the HYB 20% is maintained during 50 cycles. Moreover, the capacity retention of the hybrids is better than that of the reference so that the delivered capacity of the HYB 20% surpasses the one of the LCO reference after 40 cycles. Therefore, instead of degrading the performances of the electrode, the addition of our PTMA/C composite to LCO cathodes improves the cycle life. These results proved that the huge capacity drop observed in the case of the NMC523 hybrid cathode cannot be explained by a cobalt oxide contribution.

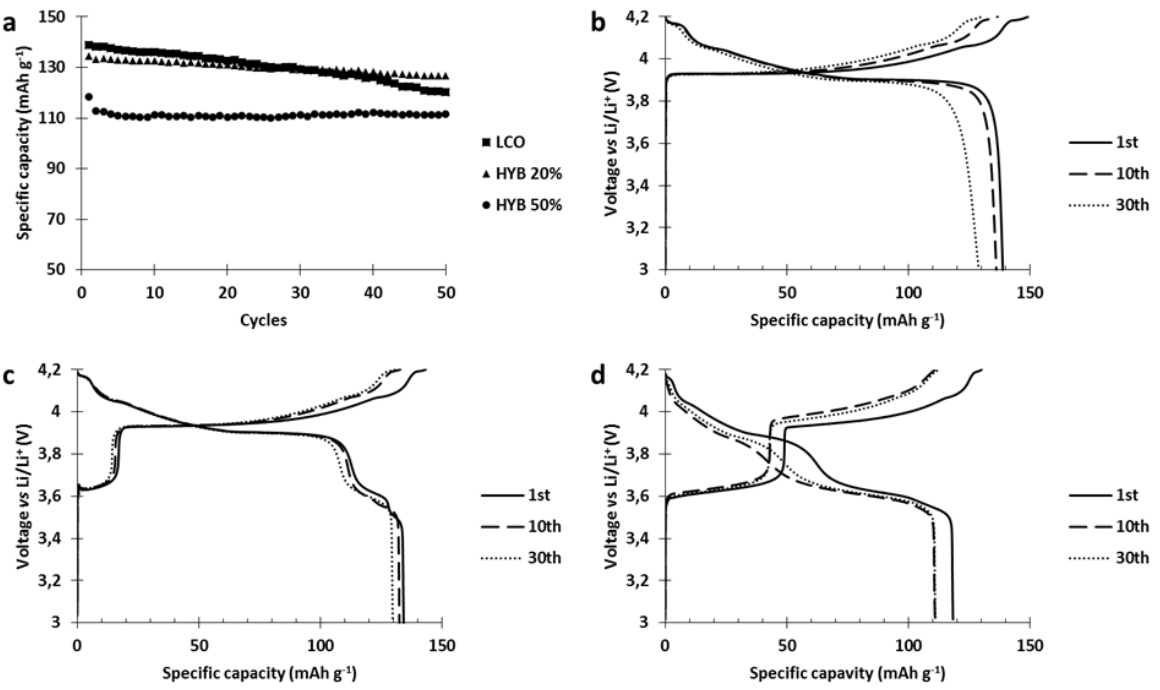

Figure S9. (**a**) Capacity retention through cycles of LCO and hybrid LCO-PTMA cathodes at C/5 (charge and discharge). (**b**) Voltage curves of LCO. (**c**) Voltage curves of Hybrid 20%. (**d**) Voltage curves of Hybrid 50%.

In order to investigate the possible contribution of the nickel oxide in the capacity fade of NMC hybrid cathodes, we have blended the spinel $LiNi_{0.5}Mn_{1.5}O_4$ (LNMO) and our PTMA composite (50 wt%). A single constituent LNMO reference was produced in the same way (aqueous process) and the cathodes were cycled at C/5 in a half cell setup between 3.4 and 5 V vs. Li/Li^+ (Figure S10). The initial capacity delivered by the LNMO reference cathode is only of 80 mAh g^{-1} and, after 10 cycles, stabilizes around 120 mAh g^{-1}. The 110 mAh g^{-1} capacity delivered by the hybrid cathode is stable through cycles. Both cathodes present a very low initial coulombic efficiency (discharge capacity/charge capacity) of 33% for the LNMO reference and 45% for the hybrid cathode. These results proved that the huge capacity drop observed in the case of the NMC523 hybrid cathode cannot be explained by a nickel oxide contribution.

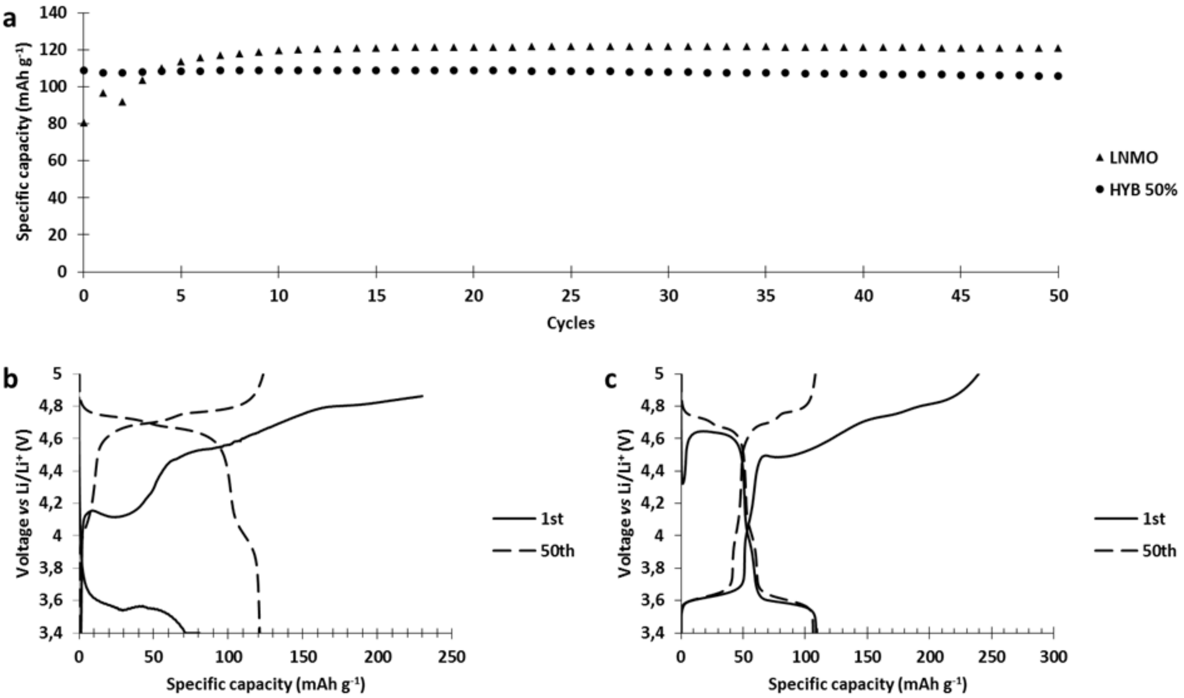

Figure S10. (**a**) Capacity retention through cycles of the LNMO reference and the hybrid cathodes cycled at C/5 (charge and discharge). (**b**) Voltage curves of the LNMO reference. (**c**) Voltage curves of the hybrid cathode.

2.3. Effect of water on NMC particles

Since water has a strong deleterious impact on hybrid cathodes containing NMC523 which is not observed with the hybrid cathodes made with the other inorganic components, we measured the pH of slurries comprising the different intercalation materials (Figure S11). The NMC523 slurry presents a highly alkaline pH of 11.3 while others remain below 10. To reach such a high pH, alkaline species like LiOH must have dissolved into water from the surface of the NMC particles.

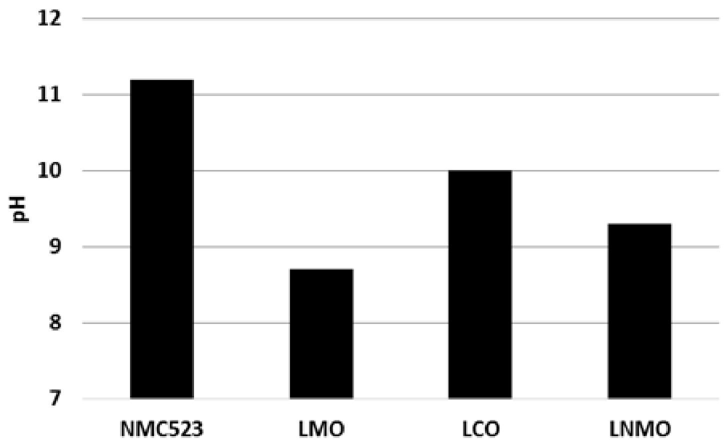

Figure S11. The pH of the different slurries monitored after 10 min of mixing. The slurries are made with 84 wt% of intercalation materials, 3 wt% SBR, 3 wt% CMC and 10 wt% C65.

3. Surface passivation of NMC811 particles

In order to give credit to our hypothesis concerning the formation of a passivation layer around NMC811 particles, X-ray photoelectron spectroscopy (XPS) and TEM experiments were performed on NMC523 and NMC811 particles first brought in contact with water and the aluminum current collector, then collected for XPS analysis (Figure S12).

XPS was performed using a SSI X-Probe (SSX 100/206) photoelectron spectrometer from Surface Science Instruments equipped with a monochromatized Al Kα X-ray source. The batteries were disassembled in a glovebox after cycling, and the electrodes were washed with DMC before transfer into the XPS chamber. All the binding energies were calculated according to the C-(C, H) component of the C 1s peak fixed at 284.8 eV. Data analysis was carried out using CasaXPS software, and the peaks were decomposed by Gaussian/Lorentzian product formula GL(15) after a Shirley-type background was subtracted. TEM images were obtained with a LEO 922 OMEGA energy filter transmission electron microscope.

In the case of NMC811 particles (Figure S12b), XPS clearly shows the presence of aluminum species on the surface of the particles (peak centered at 74 eV), likely resulting from the precipitation of aluminum-containing species resulting from the dissolution of the aluminum current collector due to the alkaline species (LiOH) released from the NMC811 particles in contact with water. This was not observed for the NMC523 particles. Those observations confirm the formation of a passivation layer around NMC811 particles as hypothesized in Figure 12 of the main manuscript, while a delithiated NMC layer would be formed at the surface of NMC523. However, the passivation layer around NMC811 particles could not be visualized by TEM (compare Figures S12b and c).

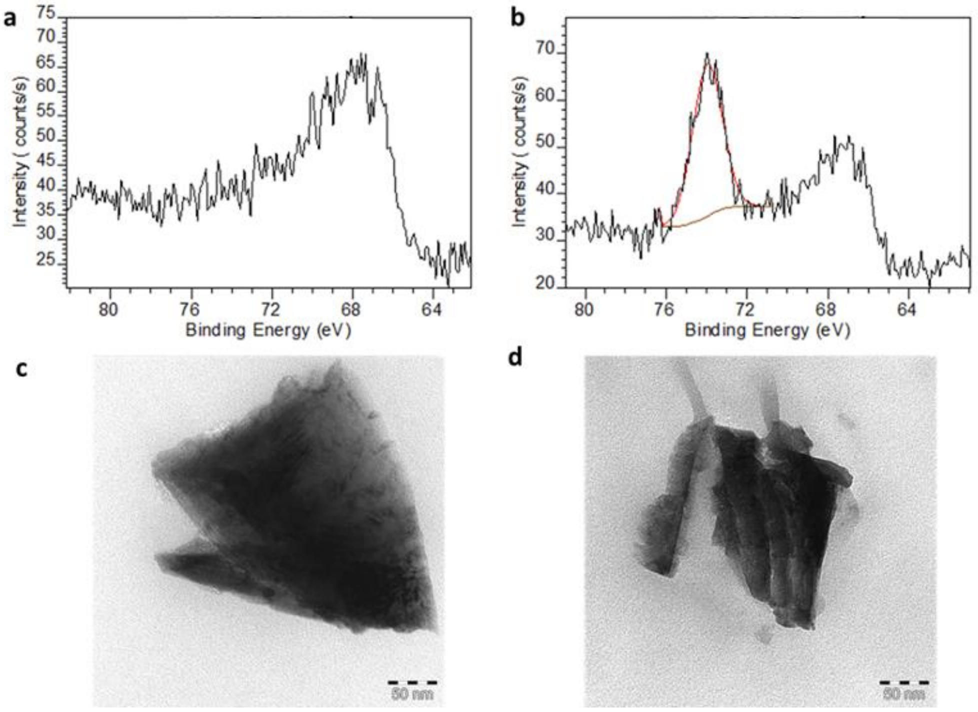

Figure S12. XPS spectra of Al_{2p} for (**a**) NMC523 and (**b**) NMC811. TEM images of (**c**) NMC523 and (**d**) NMC811.
